# The assessment of resting tongue posture in different sagittal skeletal patterns

**DOI:** 10.1590/2177-6709.24.3.055-063.oar

**Published:** 2019

**Authors:** Farheen Fatima, Mubassar Fida

**Affiliations:** 1 The Aga Khan University Hospital, Department of Surgery, Section of Dentistry (Karachi, Pakistan).

**Keywords:** Tongue, Jaw relationship, Dental arch

## Abstract

**Introduction:**

Resting tongue posture affects the surrounding structures and, theoretically, may result in altered arch form and jaw relationship.

**Objective:**

The objective of the present study was to investigate the association between resting tongue posture as observed in lateral cephalometric radiograph, sagittal jaw relationship and arch form.

**Methods:**

The study was conducted on pretreatment lateral cephalograms and dental casts of 90 subjects. Subjects were equally divided into three groups, based on sagittal jaw relationship (Class I, II and III). Tongue posture was determined in terms of tongue-to-palate distances at six different points (distances 1 to 6) using ViewPro-X software, according to the method described by Graber et al in 1997. The arch widths (intercanine and intermolar widths) were evaluated on pretreatment dental casts.

**Results:**

Tongue-to-palate distances were found to be comparable among different study groups. Significant differences were found in intercanine and intermolar widths at the cuspal and gingival levels among the study groups, except for intercanine width at cuspal level in maxilla and intermolar width at cuspal level in mandible. Moderate positive correlation was found between arch widths ratios at distances 3 and 4 in skeletal Class III group. Effect size was found to be moderate to large in different sagittal skeletal patterns and arch widths.

**Conclusion:**

The results of the current study showed no significant differences in the resting tongue posture among the groups, and moderate to weak correlation between tongue posture and dental arch widths.

## RESUMO

**Introdução:**

a postura da língua em repouso pode afetar as estruturas adjacentes e resultar em mudanças no formato das arcadas e na relação maxilomandibular.

**Objetivo:**

o presente estudo teve como objetivo avaliar a associação entre a postura da língua em repouso (observada em radiografias cefalométricas laterais), o padrão esquelético e o formato da arcada.

**Métodos:**

a amostra incluiu radiografias cefalométricas laterais e modelos de estudos iniciais de 90 pacientes, os quais foram divididos em três grupos, conforme a relação esquelética: Classe I, Classe II e Classe III. A postura da língua foi avaliada com o software ViewPro-X, de acordo com o método descrito por Graber et al. em 1997, medindo-se a distância entre o palato e a língua em seis pontos diferentes (distâncias 1 a 6). A largura das arcadas (distâncias intercaninos e intermolares) foi medida nos modelos de gesso iniciais.

**Resultados:**

as distâncias aferidas foram semelhantes entre os grupos. Diferenças significativas entre os grupos foram encontradas para as distâncias intercaninos e intermolares, nos níveis das cúspides e da gengiva, com exceção da distância intercaninos superiores ao nível das cúspides e distância intermolares inferiores ao nível das cúspides. No grupo Classe III, foi observada uma correlação positiva moderada entre as proporções das larguras das arcadas nas distâncias 3 e 4. O tamanho do efeito apresentou-se de moderado a alto nos diferentes padrões esqueléticos e larguras das arcadas.

**Conclusão:**

os resultados do presente estudo mostraram ausência de diferenças significativas na postura da língua em repouso entre os diferentes grupos, e uma correlação moderada a fraca entre a postura da língua e o formato das arcadas.

## INTRODUCTION

The biological principles peculiar to the orthodontic practice have been fundamentally limited to the hard tissue structures, i.e., teeth and bone. A review of the orthodontic literature reveals that our perception of the soft tissue forces and equilibrium of oral musculature has remained relatively undeveloped.^1^ In most of the cases, the dental correction of malocclusion is focused towards camouflage treatment. The treatment is usually followed by retention of the achieved outcome, to allow establishment of new neuromuscular equilibrium in orthodontically established occlusion.^2^ Unfortunately this is not always the case and abnormal muscle forces may result in relapse.^3^ Hence, using an objective method for the evaluation of neuromuscular behavior may help in establishing stable occlusion.^3^

The concept of equilibrium of the labio-lingual muscular forces has been recognized by many orthodontists.^2^ ^-^ ^4^ They became aware of the role of muscles in maintaining the stability of the arch shape and position of teeth. Winders^5^ reported that the tongue exerts more pressure on the dentition than the buccal muscles. Similar results were later reported by Kydd and Neff^6^ and Kydd et al.^7^ According to them, the magnitude of muscular forces exerted by tongue during rest and function is higher as compared to lips and cheeks.^8^ ^-^ ^10^ Hence, it is inferred that tongue plays a vital role in the establishment of alveolar arch form and in positioning teeth over the basal bone. The effect of tongue size on mandibular arch perimeter has been reported by a few studies.^8^ ^-^ ^10^ Moreover, the resting tongue posture was found to be associated with sagittal jaw relationship.^11^ Lowered tongue posture was reported in skeletal Class III as compared to Class I patients.^11^

Maxillary and mandibular growth is influenced by genetic and environmental factors that affect the receptiveness and response of cells to the stimuli.^2^ Brodie^12^ believed that the alveolar bone is labile and the teeth take their position around the borders of tongue. The soft tissue forces play important role during maxillary-mandibular growth, and may influence the establishment of jaw relationship; however, the degree of its influence on the final form is still a matter of debate.^8^ ^,^ ^9^ Sagittal jaw relationship is established during adolescence period.^13^ Early preventive and interceptive treatment facilitates growth in favorable direction, but relapse may occur due to the expression of original growth pattern and abnormal muscle forces if the underlying cause remains untreated.^10^ Therefore, treatment of underlying neuromuscular imbalance can help provide stability to the achieved corrections.^12^ ^,^ ^14^

Abnormalities in either function or position of tongue can lead to changes in the surrounding dento-alveolar structures.^8^ ^-^ ^11^ Thus, considering the etiological factor before starting the orthodontic treatment may enhance the efficacy and long-term stability of treatment. A survey of pertinent literature showed that this topic encompasses conflicting reports that the variations in tongue features can influence the surrounding dentoalveolar form.^15^ ^-^ ^17^ Therefore, the aim of present study was to assess the tongue posture in different sagittal skeletal patterns. The secondary objective was to compare the arch widths, at intercanine and intermolar levels of maxilla and mandible, among various sagittal skeletal patterns.

## MATERIAL AND METHODS 

A cross-sectional study was performed on pretreatment lateral cephalograms and dental casts of patients attended at a dental clinic in 2017. A total of 250 records were evaluated and subjects matching the inclusion criteria were selected using purposive sampling technique. Ethical exemption was taken from the ethical review committee of The Aga Khan University (reference number 4640-17) prior to the data collection. Sample size was calculated in NCSS PASS (Kaysville, UT, USA) software, using the effect size (ES) assumed to be 1.00, as reported by Primozic et al,^11^ which showed that a total sample of 75 subjects was required to achieve 90% power to detect statistically significant differences with the alpha set as 0.05. To ensure the validity of comparison among the groups, the sample size was increased to 90 subjects. Subjects were equally divided into the following three study groups, based on sagittal jaw relationship:

» Class I: ANB angle 0° to 4°
» Class II: ANB angle > 4°
» Class III: ANB angle < 0°.

Equal male and female subjects were included in each group. Subjects were recruited from Pakistani population, with age range of 18 - 25 years, having good quality standardized pretreatment dental casts and lateral cephalograms with good visibility of tongue and full set of permanent dentition until second molar, with normodivergent growth pattern. Whereas, subjects with history of previous orthodontic or orthopedic treatment, presence of any craniofacial and dental anomaly, habits such as tongue thrust, thumb sucking and mouth breathing, syndromes or history of trauma and surgery involving tongue and oral musculature were excluded. 

The subjects were evaluated for tongue posture and morphological characteristics of dental arches using pretreatment lateral cephalogram and dental casts, respectively.

### Evaluation of tongue posture

**Figure 1:**
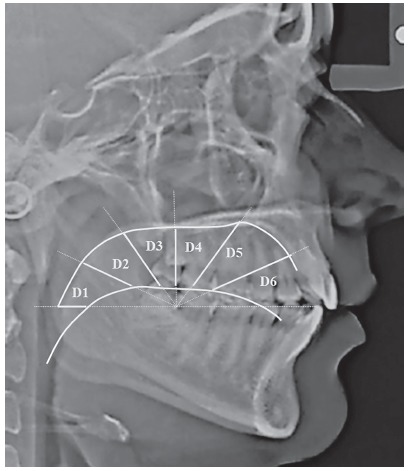
Assessment of tongue-to-palate distances on lateral cephalogram.

Pretreatment lateral cephalograms of all subjects were obtained using Orthoralix R9200 (Gendex-KaVo, Milan, Italy) at 165-cm film to tube distance. The subjects’ head were stabilized using rigid head fixation, with the Frankfurt horizontal plane oriented parallel to the floor. To obtain records in resting tongue position, the participants were instructed to swallow and then relax.^11^ Furthermore, the patients were explained about the centric occlusion and lips resting position.^11^ Tongue posture was determined using the method described by Graber et al,^14^ using ViewPro-X (Rogan-Delft, Veenendaal, Netherlands) software. A template was drawn on the lateral cephalogram with its horizontal line extending through the incisal edge of lower central incisor and the cervical distal third of lower second molar extending posteriorly. Taking cervical area as centre, angles were drawn at 30^o^, 60^o^, 90^o^, 120^o^ and 150^o^. The contours of the dorsum of tongue and the palate were traced, and six distances (D1 - D6) were recorded at 0^o^, 30^o^, 60^o^, 90^o^, 120^o^ and 150^o^ between tongue and palate contours (Fig 1).^11^

### Evaluation of dental casts

**Figure 2:**
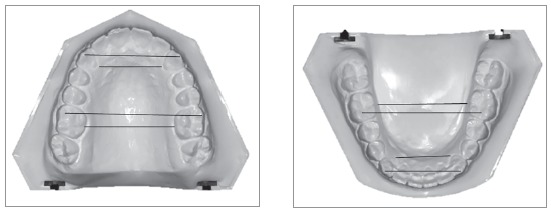
Measurement of morphological characteristics of dental arches.

Pretreatment dental casts were used to determine intercanine and intermolar widths at the cuspal and gingival levels using the digital vernier caliper (0-150mm ME00183, Dentaurum, Pforzheim, Germany) with an accuracy of 0.02 mm and a reliability of 0.01 mm according to the manufacturer’s specification. Intercanine width (IC) was measured at cusp tips and at gingival margin lingually at deepest concavity for maxillary and mandibular arches. Similarly, intermolar width (IM) at cuspal level was measured at mesiobuccal cusp tip and at lingual gingival margin at lingual grooves, as shown in Figure 2. 

### Statistical analysis

Data were analyzed using SPSS software for Windows (version 20.0, SPSS Inc. Chicago). Shapiro-Wilk test was used to check the normality of the measurements, which showed a non-normal distribution; hence, nonparametric tests were applied. Mann-Whitney U test was applied to compare the study parameters between genders, and showed nonsignificant differences; therefore, to conserve the power of study, data were not split on gender basis. Kruskal-Wallis test was used to assess the differences in tongue posture and arch widths (intercanine and intermolar widths) among the groups. The average values were calculated for intercanine and intermolar widths at cuspal and gingival level. The ratio scores were calculated as:

» IC ratio = mandibular intercanine width / maxillary intercanine width.
» IM ratio = mandibular intermolar width / maxillary intermolar width.

These ratio scores were correlated with six tongue-to-palate distances (D1 - D6) for each group, using Spearman’s correlation. To determine the magnitude of variance caused by resting tongue posture on skeletal pattern and dental arch widths, the effect size (ES) was calculated. A p-value of ≤ 0.05 was considered as statistically significant. 

## RESULTS

**Figure 3:**
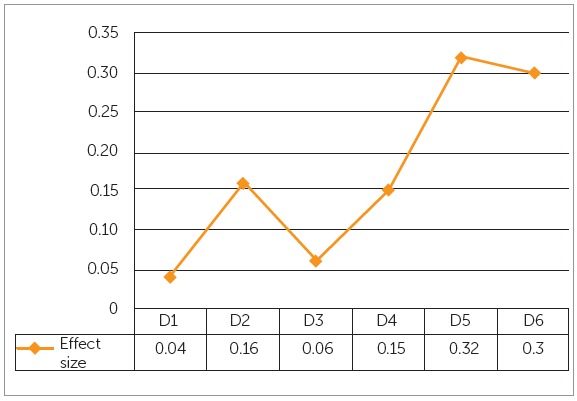
Effect size of tongue-to-palate distances at the six measurement points between skeletal classes. D = distance, small: ≤ 0.2, Medium: 0.5 - 0.7, Large: ≥ 0.8.

**Figure 4:**
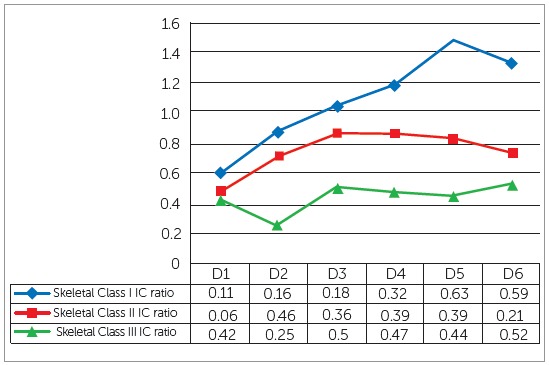
Effect size of tongue-to-palate distances at six measurement points and intercanine width ratios in different skeletal classes: D = distance, Small: ≤ 0.2, Medium: 0.5 - 0.7, Large: ≥ 0.8.

**Figure 5:**
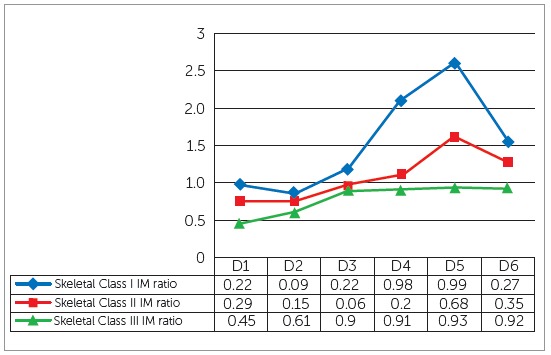
Effect size of tongue-to-palate distances at six measurement points and intermolar width ratios in different skeletal classes: D = distance, Small: ≤ 0.2, Medium: 0.5 - 0.7, Large: ≥ 0.8.

The mean age of sample was 21.26 ± 3.9 years. The comparison of mean tongue-to-palate distances among the study groups showed statistically nonsignificant differences (Table 1). Significant differences were found for arch widths at cuspal and gingival levels among the three groups, except at maxillary intercanine width at cuspal level and mandibular intermolar width at cuspal level (Table 2). Pairwise comparison of morphological characteristics among skeletal patterns is shown in Table 3. The correlation between arch width ratios and skeletal classes is shown in Table 4. Moderate positive correlation was found at D3 and D4 in skeletal Class III group. Magnitude of alteration in skeletal pattern and arch width ratios caused by resting tongue posture varied from moderate to large (Figs 3, 4 and 5). To estimate measurement error, 30 lateral cephalograms and dental cast were re-evaluated by the main investigator. The assessment of reliability of tongue-to-palate distances showed excellent agreement between the two sets of readings (r = 0.9 - 0.98) (Table 5). Similarly, casts were analyzed to estimate measurement error, and results showed excellent agreement between the two evaluations (r = 0.86 - 0.98) (Table 6).

**Table 1:** Comparison of tongue-to-palate distances among skeletal patterns

Tongue-to-palate distances	Skeletal Class I (n = 30) Mean ± SD (mm)	Skeletal Class II (n = 30) Mean ± SD (mm)	Skeletal Class III (n = 30) Mean ± SD (mm)	p-value
D1	3.4 ± 1.9	3.3 ± 1.8	3.7 ± 2.1	0.74
D2	4.1 ± 2.4	3.1 ± 1.5	3.4 ± 1.8	0.35
D3	5.2 ± 2.5	4.5 ± 2.0	4.8 ± 2.6	0.71
D4	7.1 ± 3.3	6.0 ± 2.9	6.9 ± 3.8	0.33
D5	9.2± 5.0	7.2 ± 4.4	8.8 ± 4.8	0.25
D6	10.9 ± 5.4	9.5 ± 5.5	8.4 ± 5.2	0.14

n = 90; SD = standard deviation, Kruskal-Wallis test, *p < 0.05; ** p < 0.001.

**Table 2:** Comparison of morphological characteristics among skeletal patterns.

Cast analysis	Skeletal Class I (n = 30) Mean ± SD (mm)	Skeletal Class II (n = 30) Mean ± SD (mm)	Skeletal Class III (n = 30) Mean ± SD (mm)	p-value
IC-cusp maxilla	31.7 ± 3.2	31.7 ± 3.6	32.8 ± 2.4	0.22
IC-cusp mandible	24.0 ± 2.8	24.5 ± 2.7	25.8 ± 3.0	0.03*
IC-gingiva maxilla	23.7 ± 2.4	23.3 ± 2.6	25.3 ± 2.9	0.01*
IC-gingiva mandible	19.1 ± 1.8	19.3 ± 3.0	20.4 ± 2.2	0.03*
IM-cusp maxilla	46.4 ± 8.1	48.0 ± 3.6	49.9 ± 3.1	0.04*
IM-cusp mandible	42.1 ± 3.4	42.6 ± 3.4	44.0 ± 3.8	0.40
IM-gingiva maxilla	34.5 ± 3.3	34.1 ± 3.2	36.2 ± 2.9	0.03*
IM-gingiva mandible	31.3 ± 2.8	32.5 ± 2.8	37.5 ± 2.7	0.02*

n = 90; SD = standard deviation, Kruskal-Wallis test, *p < 0.05; ** p < 0.001.

IC = intercanine width, IM = intermolar width.

**Table 3:** Pairwise comparison of morphological characteristics among skeletal patterns.

Parameters	Skeletal Class I vs. II p-value	Skeletal Class II vs. III p-value	Skeletal Class I vs. III p-value
IC-cusp maxilla	0.75	0.1	0.19
IC-cusp mandible	0.29	0.08	0.01*
IC-gingiva maxilla	0.38	0.005*	0.03*
IC-gingiva mandible	0.68	0.03*	0.01*
IM-cusp maxilla	0.87	0.01*	0.02*
IM-cusp mandible	0.73	0.43	0.16
IM-gingiva maxilla	0.76	0.01*	0.05*
IM-gingiva mandible	0.45	0.04*	0.01*

n = 90; Mann-Whitney U test, *p < 0.05; ** p < 0.001.

IC = intercanine width, IM = intermolar width.

**Table 4:** Association of tongue posture and morphological characteristics.

Tongue-to- palate distances	Skeletal Class I (n = 30)		Skeletal Class II (n = 30)		Skeletal Class III (n = 30)	
	IC ratio (r)	IM ratio (r)	IC ratio (r)	IM ratio (r)	IC ratio (r)	IM ratio (r)
Distance 1	0.02	-0.01	-0.13	0.21	-0.13	-0.17
Distance 2	0.14	-0.07	0.18	0.26	-0.1	-0.01
Distance 3	0.09	-0.18	-0.04	0.05	0.47	0.3
Distance 4	0.2	-0.04	0.01	0.05	0.31	0.4
Distance 5	0.23	-0.01	-0.04	0.03	0.33	0.17
Distance 6	0.08	-0.05	-0.12	0.14	0.24	0.22

n = 90; Spearman’s correlation (r), IC = intercanine width, IM = intermolar width.

P value > 0.05, r = 00 - 0.19 ? very weak, 0.20 - 0.39 ? weak, 0.40 - 0.59 ? moderate, 0.60 - 0.79 ? strong, 0.80 - 1.0 ? very strong.

**Table 5:** Assessment of the reliability of measurements.

Tongue-to-palate distances	First reading (n = 30) Mean ± SD (mm)	Second reading (n = 30) Mean ± SD (mm)	ICC
D1	3.4 ± 1.9	3.6 ± 1.7	0.97
D2	4.1 ± 2.4	4.0 ± 2.3	0.98
D3	5.2 ± 2.5	5.1 ± 2.4	0.98
D4	7.1 ± 3.3	6.9 ± 3.3	0.98
D5	9.2± 5.0	8.7 ± 5.0	0.98
D6	10.9 ± 5.4	10.2 ± 5.5	0.97

n = 30; SD = standard deviation, D= distances. ICC = Intraclass correlation coefficient.

ICC: > 0.75 excellent agreement, 0.4 - 0.75 fair agreement, < 0.4 poor agreement.

**Table 6:** Assessment of the reliability of measurements.

Cast analysis	First reading (n = 30) Mean ± SD (mm)	Second reading (n = 30) Mean ± SD (mm)	ICC
IC-cusp maxilla	31.7 ± 3.2	32.1 ± 3.1	0.98
IC-cusp mandible	24.0 ± 2.8	24.3 ± 3.6	0.86
IC-gingiva maxilla	23.7 ± 2.4	24.3 ± 2.5	0.97
IC-gingiva mandible	19.1 ± 1.8	19.4 ± 1.9	0.97
IM-cusp maxilla	46.4 ± 8.1	46.2 ± 8.1	0.89
IM-cusp mandible	42.1 ± 3.4	42.4 ± 3.3	0.98
IM-gingiva maxilla	34.5 ± 3.3	34.4 ± 3.7	0.94
IM-gingiva mandible	31.9 ± 2.8	31.9 ± 3.0	0.89

n = 30; SD = standard deviation, IC = Intercanine width, IM = Intermolar width. ICC = Intraclass correlation coefficient.

ICC: > 0.75 excellent agreement, 0.4 - 0.75 fair agreement, < 0.4 poor agreement.

## DISCUSSION

Light forces exerted by perioral muscles are considered to be more important than the intermittent forces during oral function (speech, swallowing and mastication).^18^ The forces exerted by tongue play an important role in the guidance of tooth eruption, dental arch form and stability.^8^ ^-^ ^10^ The objective of present study was to assess the resting tongue posture in various sagittal skeletal patterns. The resting tongue posture of skeletal Class II patients was found to be higher as compared to Class I and III subjects. Meanwhile, lowered tongue posture was observed in posterior most area in skeletal Class III subjects. Comparable results were reported by Primozic et al,^11^ who conducted a case-control study between Class I and III individuals, and found lowered tongue posture in Class III individuals. Similar results were reported by Guay et al^19^.

The transverse characteristics of dental arches were observed as intercanine and intermolar widths at cuspal and gingival levels. The mean values were found to be higher in skeletal Class III group, as compared to Class I and II groups. Previous study reported higher mean intermolar widths in maxilla and mandible, and intercanine width in mandibular arch in Class III, as compared to Class I subjects.^11^ Increased arch widths were found in maxillary arch in Class III individuals. The possible reason of increased maxillary intercanine width could be to compensate the outgrowing mandible, to camouflage the true transverse skeletal discrepancy. Significant differences were found in intercanine and intermolar widths among the groups, except at intercanine width at cuspal level in maxilla and intermolar width at cuspal level in mandible. The plausible cause of nonsignificant differences in these areas may be the dental compensations to camouflage the skeletal malocclusion.^20^ ^,^ ^21^

The position of teeth on dental arch is affected by surrounding pressure from lips, cheeks and tongue.^15^ ^-^ ^17^ ^,^ ^22^ Therefore, the altered position of tongue may cause imbalance in the forces, which may result in alteration in the dental arch form. Tongue posture was found to be very weak or weakly correlated to dental arch width in skeletal Class I and II. Moderate correlation was found at D3 and D4 in skeletal Class III subjects at intercanine and intermolar width ratios, respectively. Previous studies reported associations between the transverse characteristics of dental arches and tongue size^23^ ^-^ ^26^ and posture.^19^ ^,^ ^27^ The results were in concordance with the study conducted by Primozic et al.^11^ These results may be the outcome of dentoalveolar compensation, which supported the idea that the postnatal development of the dental arch form is not significantly altered by variations in the resting tongue posture.^28^

Despite conflicting reports, it is generally assumed that the alveolar bone responds to external influences.^29-31^ In this regard, effect size - which is a quantitative measure of the magnitude of variance - may provide useful information regarding the assessment of tongue posture on various skeletal patterns. The results revealed that the posterior part of tongue was 4% (D1) and 16% (D2), middle portion showed 6% (D3) and 15% (D4), whereas anterior part of tongue displayed large variations in different sagittal skeletal patterns (D5 = 32%, D6 = 30%). According to these results D5 and D6 could provide reliable information about the possible discrepancies in the sagittal skeletal pattern. Another study conducted on Class I and III subjects reported large variation at D2, D3, D4 and D6.^11^

Similarly, effect size was obtained for tongue posture and dental arch widths in skeletal classes. The results revealed that moderate changes may occur in intercanine width ratio in skeletal Class I and II at D1; whereas, the other distances displayed large effect of tongue posture on intercanine width ratios in skeletal classes. Moreover, large effect of tongue posture was found at intermolar width ratios in all study groups. Although weak correlation was found between tongue posture and dental arch widths, the effect size indicated that altered tongue posture may result in variations in dental arch form.

Results of the present study suggested that although the tongue posture was not found to be significantly different in various skeletal patterns, moderate to large variation may occur in sagittal skeletal relationship with varying tongue posture. Therefore, as a clinical inference, monitoring tongue posture during interceptive and comprehensive orthodontic treatment may help in providing long term stability to the achieved results.

The limitation of present study was the use of two-dimensional imaging technique for the evaluation of tongue posture. Furthermore, muscle forces and volume of tongue are important factors that may affect the skeletal pattern and arch form, and were not taken into account in this study. With contemporary technological advancement, use of 3D CBCT and MRI may provide more information in this regard. Moreover, a cross-sectional study design may not establish a cause and effect relationship, and longitudinal studies may be required to allow more conclusive evidence. Therefore, caution should be taken in regard to the findings of the present study.

## CONCLUSIONS

1. Mean tongue-to-palate distances were found to be higher in skeletal Class III and lower in Class II subjects, compared to Class I group. However, no significant differences in tongue posture were found among the groups.
2. Mean intercanine and intermolar widths were greater in Class III subjects, as compared to Class I and II. Significant differences were found in maxillary and mandibular intercanine and intermolar widths at cuspal and gingival levels, except at maxillary intercanine and mandibular intermolar widths at cuspal level.
3. In all subjects, tongue posture was found to have moderate to weak correlation with the dental arch widths.
